# SMR Neurofeedback Training Facilitates Working Memory Performance in Healthy Older Adults: A Behavioral and EEG Study

**DOI:** 10.3389/fnbeh.2018.00321

**Published:** 2018-12-20

**Authors:** Valeska Kouzak Campos da Paz, Ana Garcia, Aloysio Campos da Paz Neto, Carlos Tomaz

**Affiliations:** ^1^Laboratory of Neuroscience and Behavior, Institute of Biology, University of Brasília, Brasília, Brazil; ^2^Department of Psychology, Euro-American University Center (UNIEURO), Brasília, Brazil; ^3^Laboratory of Neuroscience and Behavior, CEUMA University, São Luis, Brazil

**Keywords:** neuromodulation, neurofeedback, brain stimulation, working memory, aging

## Abstract

Cognitive aging has become a major concern because life expectancy has increased and elderly populations are socially and economically active. Neurofeedback is a technique of neuromodulation through operant conditioning paradigm that uses a computer interface to provide real-time information about brain activity to increase individual self-perception and assist in modulation. The sensorimotor rhythm (SMR) training protocol is known to enhance attention and has been applied to improve cognitive performance, primarily for attention and memory gains. The aim of this study is to test if the SMR protocol can improve working memory performance in an aging population and consequently favor cognitive reserve. Seventeen older adults (12 females) took part in a randomized placebo-controlled study. They completed a visual working memory test, Delayed Matching to Sample Task (DMTS), before and after the SMR neurofeedback protocol in order to compare their visual working memory performance. Moreover, a 19-channels EEG was collected while they perform the DMTS pre- and post-training. The experimental group showed an improvement in their working memory performance after the training with similar activation power, mainly in theta and beta frequency band at frontal and alpha at temporal regions. The sham group showed some variations in the score of working memory after the training, but were not statistically significant and their power spectrum demonstrate enhancement in alpha and beta band frontal and temporal. The group that did not receive neurofeedback training did not show a change in their working memory performance, neither in their EEG spectrum. The results suggest that neurofeedback can benefit brain reserve in an aging population because individuals enhanced their working memory performance after training and have their EEG activation changed according to expected in working memory tasks.

## Introduction

Cognitive aging has become a major social concern because life expectancy has increased (Baltes and Lindenberger, 1997). To preserve cognitive reserve in aging populations, research in cognitive training increased (Baltes et al., 1989), and researchers have developed tools favoring cognitive maintenance. It is known that there is a positive correlation between fluid intelligence and cognitive reserve, as well as, executive function, mainly in working memory, attention and information processing (Craik and Bialystok, 2006). However, natural changes expected in cognitive aging also affect these capabilities; therefore, these changes can be a burden to a population that is still socially active. To counteract these changes, neurofeedback, a form of neuromodulation in which individuals have information about their neurological state and are able to self-regulate their brain activity through an operant conditioning paradigm, may be a technique to preserve cognitive reserve.

During neurofeedback training, an individual receives, through a computational interface, real-time visual and/or audio information about their brain wave activity as feedback after achieving a goal. Moreover, neurofeedback training works dynamically in the cortex, that is, it can induce the individual to increase the rhythm or amplitude of a specific frequency range in the cortex; likewise, it can inhibit the rhythm or amplitude of another frequency range, either in the same training protocol or during separate training when the parameter is electrical brain activity. Accordingly, the cognitive model of Lacroix (1986) provides a broader view of neurofeedback training; it suggests that brain modification occurs not only through operant conditioning feedback but also by the modification of the individual’s perception of his physiological state, thus promoting a cognitive integration of the conditioned behavior. Therefore, there are two processes involved with neurofeedback: one is unconscious by operant conditioning and the other is conscious cognitive self-perception.

One of the firsts protocol that evaluated the association between operant conditioning and brain activity was performed by Sterman et al. (1970), in which cats trained to increase activation at 12–15 Hz in the sensory motor area were shown to be resistant to hydrazine, a convulsive compound. Afterwards, they successfully tested this protocol in humans with seizure disorders to diminish seizures, this frequency band is known as sensorimotor rhythm (SMR).

Thereafter, Lubar and Lubar (1984) tested the same protocol to increase SMR in hyperkinetic children with ADHD and demonstrated that this protocol was able to reduce excessive motor movements and increase attention.

The circuitry of SMR is a thalami-cortical, bottom-up mechanism that reduces the interference of somatosensory information, as the motor activity can interfere in information processing that results in diminished cognitive performance (Kober et al., 2015). Therefore, this inhibition, driven by the increase in SMR, leads to a greater integration of information processing in the cortex, and the SMR neurofeedback training acts within the inhibitory mechanism of the thalamic circuitry (Egner and Gruzelier, 2004). Although, Kropotov (2009) proposed that SMR is part of the alpha rhythms, similar to mu activation as thalami-cortical desynchronization with eyes opened.

Additionally, neurofeedback has been used to neuromodulate psychiatric and neurological conditions such as epilepsy, anxiety, depression and addiction (Hardt and Kamiya, 1978; Sterman, 1996; Thompson and Thompson, 1998; Hammond, 2005), and most were treated using SMR training (see Monastra et al., 2005).

Bazanova and Aftanas (2010) stated that alpha rhythm is characterized by individual differences and consequently a more effective training is based on individualized design. As only 75%–80% of the neurofeedback standard protocol has demonstrated effectiveness. Moreover, Bazanova et al. (2018) compared a standard, individualized and EMG-individualized training for ADHD in theta/beta ratio neurofeedback and have demonstrated that individualized frequency band training might improve efficiency in neurofeedback, as well as, the control of eye movement during training.

Cognitive training by neurofeedback in a healthy population with the aim to enhance performance is a recent concept and has achieved success in younger populations (Vernon et al., 2003; Gruzelier, 2009, 2014). However, there is a lack of this training in elderly populations (Angelakis et al., 2007; Lecomte and Juhel, 2011; Becerra et al., 2012; Wang and Hsieh, 2013; Reis et al., 2016).

The studies of neurofeedback training for the elderly have proposed diverse protocols with different results; however, they have been able to demonstrate that most of the cognitive effects observed are in working memory and attention, aspects directly linked with executive functions (Diamond, 2013), and whose decline is observed during healthy cognitive aging (Park and Reuter-Lorenz, 2009). Thus, these results reinforce that neurofeedback can be an important technique to preserve cognitive reserve during aging (Valenzuela, 2008; Lustig et al., 2009).

Studies of neurofeedback training in healthy, young populations have indicated that the standard SMR protocol might be an efficient method to increase semantic working memory, improve attention and perceptive ability; reduce reaction times and errors by commission (Vernon et al., 2003). On the other hand, studies on cognitive aging have highlighted the need for new instruments, technologies and tools that benefit the protection and preservation of brain activity, as its decline compromises quality of life and increases risk factors for dementia (Lustig et al., 2009). Therefore, the neurofeedback protocol presented in this article was designed to enhance cognitive performance in aging people.

## Materials and Methods

### Participants

Seventeen healthy participants (12 females, mean ± SD age: 69.05 ± 2.1) were recruited from the local community and participated in the study after having given written informed consent. The study was approved by The Brazilian National Health Committee and the University of Brasilia Ethics Committee. Subjects were randomly assigned to either a Neurofeedback training (NF) group (*n* = 7), Sham Neurofeedback training (SNF) group (*n* = 6) or No Neurofeedback training (NNF) group (*n* = 4). All subjects were screened for cognitive and psychological conditions using the Philadelphia Brief Assessment of Cognition-PBAC (Pereira et al., 2012) with the screening criterion for normal range >49; the Beck Depression Inventory-BDI with the screening criterion for normal range <13; and the Beck Anxiety Inventory-BAI (Beck, 2001) with the screening criterion for normal range <10, which showed cognitive and emotional abilities were at normal levels (Table 1).

**Table 1:** Mean results with standard error for cognitive and emotional abilities of participants.

	Mean (SE)	
	Pre-training	Post-training
Age	69.05 (±2.1)	
PBAC	54.6 (±0.97)	
BAI	7 (±1.3)	4.8 (±0.8)
BDI	9.05 (±1.6)	6.9 (±1.05)

*(1) PBAC, Philadelphia Brief Assessment of Cognition; (2) BAI, Beck Anxiety Inventory; (3) BDI, Beck Depression Inventory*.

### Design and Procedure

**Figure 1:**
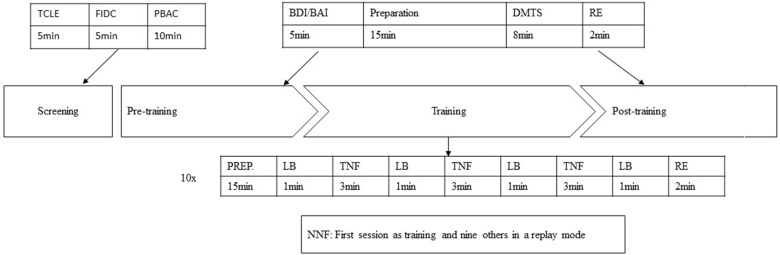
Protocol description of pre- and post-training for the NF and SNF. (1) TCLE, Brazilian free form of consent to participate in research; (2) FIDC, Record of Clinical and Demographic Information; (3) PBAC, Philadelphia Brief Assessment of Cognition; (4) BDI/BAI, Beck Depression and Anxiety Inventory; (5) DMTS, Delayed Matching to Sample Task; (6) Baseline; (7) NF, neurofeedback training; (8) SNF, sham neurofeedback training.

The design and procedure of the present study are illustrated in Figure 1. All participants were required to fill out the informed consent form, answer a questionnaire concerning demographic and clinical histories, perform a cognitive and emotional screening (PBAC, BDI and BAI) and performed a computational Delayed Matched to Sample Task (DMTS). The electroencephalographic record was made simultaneously. The participants were distributed randomly in one of the three groups. The NF started on the following day, and subjects were assigned the protocol twice a week for 5 weeks. The SNF training had the same design as the NF, but only for the first session. The other nine sessions consisted of replaying the mode of their first training. That is, they came to the laboratory in the same frequency of experimental group, however, they repeated the first session recorded by the software. The NNF had no NF training at all. They only performed the DMTS with a 5 week interval between tests.

A second DMTS computational task was scheduled for the NF and SNF groups after the training period, where they also retook the BDI and BAI to check whether there were emotional changes.

#### Delayed Matching to Sample Task (DMTS)

The working memory task chosen for this study was visual because the neurofeedback protocol refers to increased attention, and this component of working memory is more evident on a visuospatial sketchpad because storage of visual and spatial information demands attention in order to hold the perception of the object for searching or prehension (Baddeley, 2003). Moreover, the attentional disrupt process of spatial information is different from phonological information, that is, while phonological information overloads according to the amount of information given, spatial information overloads according to time delay between information and its use (Vogel et al., 2006; Luck and Vogel, 2013).

According to that, the DMTS task (Stromer et al., 1993; Holdstock et al., 1995; Tavares and Tomaz, 2002) a well know visual working memory test, applied to animal and human experimental design, was chosen to evaluate performance comparison. Basically, it consists in two phases, the first was a presentation of a visual stimulus on a screen followed by an interval with a gray screen. During the second phase, two stimuli were presented: the previous one and a new one. The objective of the task was to point to the first stimulus (Garcia et al., 2011).

During the present study, DMTS was modified by the researchers based on the assumption that it would overcome the ceiling effect, thus allowing a comparison of performances as repeat measures. Therefore, the delay between images needed to be higher to disrupt the attentional processes that keep the information online at the central executive level during working memory tasks (Baddeley, 2003; Vogel et al., 2006). To this aim, a pilot study was conducted, with 10 healthy adults (five females, mean ± SD age: 28.5 ± 1.9), to evaluate the best inter-trial delay time needed to disrupt performance and favor the testing of the possible facilitating effects of NF training. Four different time intervals were tested (3,000 ms, 5,000 ms, 9,000 ms, 15,000 ms), with 48 stimuli distributed randomly in 24 pairs of natural landscapes images (neutral content). The results of response time (RT) were for each condition: 1.15s (3,000 ms), 1.19 (5,000 ms), 1.32 (9,000 ms) and 1.35 (15,000 ms) and the performance were 92% (3,000 ms), 100% (5,000 ms and 9,000 ms) and 80% (15,000 ms). The non-parametric Wilcoxon test between RT and performance have demonstrated a significant difference (*p* < 0.001) between these two factors in all condition. Therefore, the delay of 15,000 ms induced more disrupting effects and consequently more errors. Thus, it was used as the inter-trial delay in this study.

**Figure 2:**
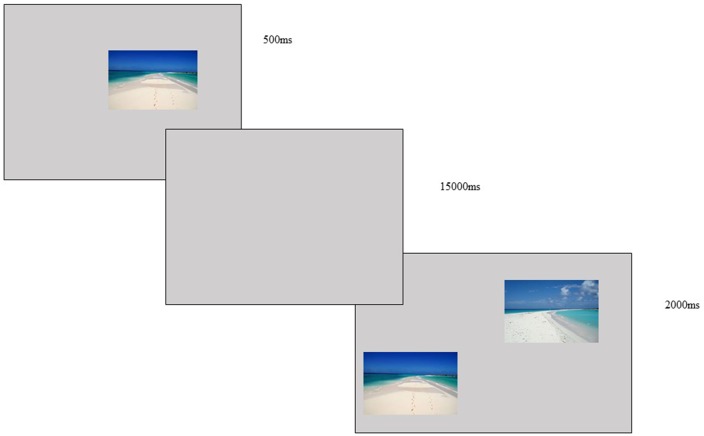
DMTS task—image presented for 500 ms, with a delay of 15,000 ms and two images presented for 2,000 ms, the subject was asked to choose the image first presented and use a mouse to click on the correct choice.

The DMTS task of the study consisted of two phases. In the first one, the participant saw an image (5 × 5 cm) in the center of a computer screen (19 inch) for 500 ms followed by an interval of 15,000 ms (gray screen). In the second phase, two images (5 × 5 cm each) were presented for 2,000 ms in which one of them was the previous image and the other a new image. The participant was asked to choose the familiar image (the one that was presented in the first phase) by clicking on it using a computer mouse. A sharp audio feedback was given for correct responses and a bass sound was given for incorrect and/or missed responses. The images consisted of natural landscapes selected from free databases on the internet (neutral visual content). They were presented as 48 stimuli arranged in 24 different pairs (see Figure 2).

#### EEG Data Acquisition and Processing

During the DMTS task an electroencephalographic data were collected by a 19 channels cap (Waveguard connect, Berlin-Germany) placed in the scalp according to the international 10/20 system and two references electrodes on the right and left ear lobe. The records were taken simultaneously at a sampling rate of 2,000 Hz with an analog band-pass filter of 0.01–100 Hz using NeuronSpectrum-4/EP system (Neurosoft, Russia). Input impedances were maintained under 5 kΩ during the whole session. All data were processed using customized Matlab scripts built to digitally separate in to non-overlapping epochs time-locked to the task condition. EEG data were analyzed using the open source EEGLAB toolbox, version 9.0.4.5 (Delorme and Makieg, 2004,^1^). These epochs were submitted to an infomax algorithm to decompose into their independent components (ICAs; Delorme et al., 2007). The components related to eye movement or blinking were removed from the original data, and the record was recalculated using the remaining components filtered and processed for extraction of measures, also with customized Matlab scripts. The pre-computed data were calculated in spectrum power and displayed for analysis in traditional frequency band: theta (4–8 Hz), alpha (8–13 Hz), beta (13–30 Hz) and gamma (30–70 Hz). Before and after the DMTS, eyes closed baseline was collected to provide an EEG rest information. Those records were extracted from the data before analysis, as the objective was to evaluate DMTS performance.

#### Neurofeedback Training

The NF and the SNF were given 10 training sessions conducted twice a week for five consecutive weeks.

The equipment employed for neurofeedback training consisted of a ProComp Infiniti differential amplifier and BioGraph Infiniti software (Thought Technology Ltd., Montreal, QC, Canada). An electrode was placed at the central area (Cz) according to the international 10/20 system. A reference and ground were placed on the left and right earlobes respectively, with input impedance under 5 kΩ. An elastic band respiratory sensor with one electrode was used to measure respiratory frequency, and a blood volume pulse electrode was used for heart rate measurement.

The training protocol consisted of 1-min baseline and 3 min of neurofeedback distributed in three blocks. The amplifier pattern of the ProComp sampled the raw EEG at 256 Hz and converter A/D for online feedback. The software applied an Infiniti Impulse Response (IIR) filter to the recorded signal to extract frequency domain information. Spectral amplitude estimates were calculated for the active site (Cz) on raw 1-s EEG segments. A bandpass filter was used to extract the reward EEG frequency band for SMR (12–15 Hz), and feedback was given when the participant increased their SMR (12–15 Hz) by 10% for each baseline measured. Visual feedback was provided in the form of pictorial image animation. There was also a respiratory pacer of six cycles per minute, and the wave of cardiac and respiration frequency was measured to provide physiological data of Heart Rate Variability (HRV). This was done because the resonant frequency respiration training interfered with the somatic system action (Lerher and Gavirtz, 2014), influencing the bottom-up circuitry that produced the SMR (Reid et al., 2013). The physiological data were not used as measure for feedback. The feedback was provided when individuals achieve the threshold established by the EEG-SMR as described above.

Although, the subjects were instructed to not move during the training, keep calm and relaxed. Motor and theta activity were not controlled. Therefore, the subjects could use any strategy to achieve the training goal.

The SNF had their first session recorded as the NF, from second to 10th session, they watched their own first training in a replaying mode provided by the BioGraph Infiniti software. They were unaware of their sham condition.

### Statistical Analysis

To analyze the behavioral results according to the mean number of correct responses during DMTS pre- and post-training, a comparison between subjects according to groups was conducted using the parametric *t*-test for paired sample.

Moreover, to analyze groups by condition, an analysis of variance (ANOVA) was conducted for all subjects with a *post hoc* Bonferroni correction. All statistical analyses were conducted using BioStast 5.0 software (Mamirauá Institute, Pará, Brazil) with statistical significance of *p* < 0.05 for a confidence interval of 95%.

The comparison of spectrum power EEG was conducted by ANOVA with a *post hoc* Bonferroni correction between groups. Moreover, the comparison pre- and post-training was conducted by the parametric *t*-test for paired sample (between subjects) and non-paired sample (between groups) processed by EEGlab, also with statistical significance of *p* < 0.05 for a confidence interval of 95%.

## Results

### Behavioral Results

**Figure 3:**
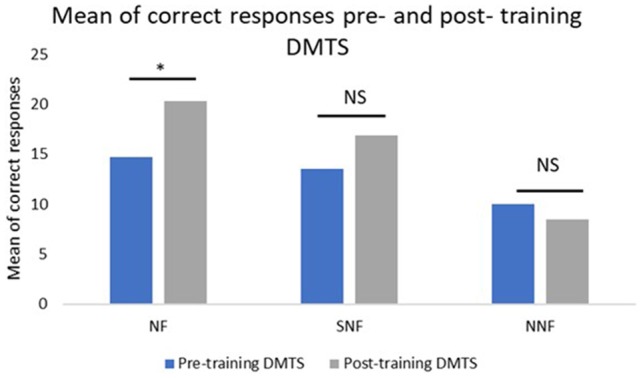
Mean number of correct responses pre- and post-training, where the NF group showed a significant difference between the conditions, while the SNF, although increasing their performance, had no statistically significant difference between conditions. The no neurofeedback training (NNF) had no changes in their performance. ^*^Significant difference; NS, not significant.

For the NF group, the mean number of correct responses at pre-training was 14.57, while the mean at post-training was 20.14. The difference between sessions was 5.57. The *t*-test for paired sample indicated a *p* = 0.0024. For the SNF group, the mean number of correct responses at pre-training was 13.5, while at post-training it was 16.83. The difference between sessions was 3.33. The *t*-test for paired sample indicated a *p* = 0.1877. For the NNF group, the mean number of correct responses at pre-training was 10, while at post-training it was 8.5. There was a difference of −1.5 with a *p* = 0.3189 (Figure 3).

**Figure 4:**
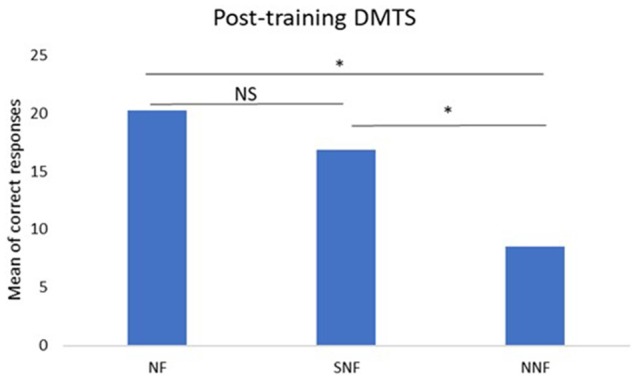
Mean number of correct responses in the post-training DMTS test. ANOVA and *post hoc* Bonferroni tests indicate significant differences between NF and NNF groups, and between SNF and NNF groups, but not between NF and SNF groups. ^*^Significant difference; NS, No Significance.

The analyses between groups using ANOVA for the pre-training condition did not show significant differences between the NF, SNF and NNF groups (*p* > 0.05). However, the ANOVA analysis did show a significant difference between groups for the post-training condition, with *p* = 0.0021, *F* = 10.25. The Bonferroni *post hoc* test indicated differences between NF and NNF (*p* < 0.05), and SNF and NNF (*p* < 0.05), with no difference between SNF and NF (Figure 4).

### EEG Results

The comparison between groups by the ANOVA demonstrated significant difference at pre-training for the frequency range of theta, alpha, beta and gamma. However, it is observed that the spectrum of the NNF group had a lower intensity compared to other groups as it had less subjects, therefore it can be inferred that the difference in the number of subjects between groups can have influenced the results, even with statistical correction. However, at the post-training condition the ANOVA between groups also demonstrated difference in all frequency range, but in less regions of the cortex compared to the pre-training condition. Therefore, at the post-training condition, it can be observed statistical difference in the frequency range of theta at left occipital (O1), alpha frequency at left central area (Cz, C3 and T3) and occipital (O1); beta frequency at the central region (Cz and C3) and occipital (O1); and gamma difference was at the frontal area (F8).

**Figure 5:**
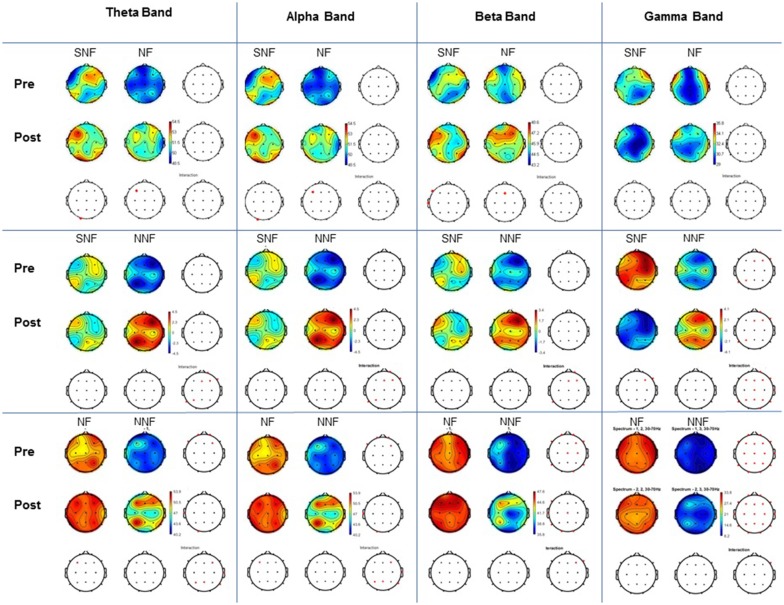
Comparison between groups and condition. For each frequency band examined (Theta, Alpha, Beta and Gamma) shown in the columns, pre and post conditions are indicated in the horizontal blocks (rows). All paired comparisons for each group (SNF vs. NF, SNF vs. NNF, and NF vs. NNF) are shown in the corresponding wave bands columns. *T*-test with Bonferroni correction and interaction, with *p* < 0.05.

The comparison between groups and condition pre- and post-training (Figure 5) demonstrated for theta band statistical differences at the pre-training between NF and NNF groups at and frontal area (Fp2, Fp1, F4 and F7). At the post training also between NF and NNF groups at temporal (T3) and occipital (O1) area. On the other hand, the comparison between subjects pre and post training demonstrated statistical differences in SNF (O1) and NF (F3). For the alpha band results of the comparison were exactly the same as for theta band between groups and between subjects.

The comparison for beta band demonstrated statistical differences at the pre-training condition between NF and NNF in more regions including frontal (Fp1, Fp2, F4, F7 and F8), central (Cz and C4), temporal (T5 and T6) and occipital (O2). At the post-training condition differences were also between NF and NNF at frontal (F8), central (Cz), temporal (T3 and T5) and occipital (O1 and O2). The comparison between subjects at pre- and post-training demonstrated statistical differences between SNF (F7 and T3) and NF (Fz).

For gamma band there were statistical differences at the pre-training analysis between groups at SNF and NNF at right frontal (Fp2, Fz, F4 and F8) and left parietal (P3) and temporal (T5), as well as, NF and NNF for almost all placements except (T3), and at the post-training condition the same groups demonstrated statistical differences in the same regions. The comparison between subjects at pre- and post-training condition demonstrate difference only for SNF at the right frontal (F4 and F8) region.

## Discussion

The aim of this study was to evaluate the effects of neurofeedback training on working memory performance in a healthy aging population. The results show that the participants who had neurofeedback training increased their performance on a visual working memory task compared to participants who did not have training. That is, the individuals of the NF group achieved a higher number of correct responses after the neurofeedback training in comparison to the SNF group and an even higher number of correct responses in comparison to the NNF group (Figure 3). This suggests that neurofeedback favors the attentional processes critical to good performance in working memory tasks (Engle, 2001; Kane and Engle, 2002; Cowan et al., 2005; Cowan and Morey, 2006).

Comparison between groups before training showed their performances to be similar (*p* > 0.05 ANOVA). That is, there were no differences between them previous to their exposure to neurofeedback that could interfere with their performance on the DMTS. Moreover, the comparison between the groups in the post-training condition showed a higher performance of the individuals from the NF group compared to the NNF group, indicating that neurofeedback training was responsible for the working memory enhancement (Figure 4).

Becerra et al. (2012) conducted a neurofeedback protocol in aging populations using the suppression of theta waves who present abnormally absolute theta power evaluating general cognition by WAIS-III and neuropsychological aspects by NEUROPSI. Their results indicated that, aside from an improvement in verbal comprehension and general IQ in WAIS-III, an improvement in working memory in the NEUROPSI test was also achieved. This result is related to increases in theta activity and highlights the increase of working memory performance in neurofeedback training.

Wang and Hsieh (2013) conducted a study comparing elderly and young populations using neurofeedback training in order to investigate changes in working memory. Their protocol’s main goal was to increase theta activity in the frontal medial area, according to Klimesch (1996, 1999). This training favored working memory enhancement. Their results showed an improvement in attention and working memory on the Attention Network Test (Fan et al., 2002) and Modified Sternberg Recognition Test (Sternberg, 1966) for both populations.

The present study tested the SMR protocol considering the attentional processes at the central executive level, which, according Vernon et al. (2003) who tested the SMR (Monastra et al., 2005) and Theta protocols (Klimesch, 1999) for cognitive enhancement in the central area of the cortex, have demonstrated that the group who underwent the SMR protocol have shown better results in attention and working memory tests than the theta groups.

Therefore, the results presented in this study reinforce the idea that neurofeedback improves not only attentional processes, but also working memory. This is because the central executive working memory can be associated with the Supervisory Attentional System as stated by Norman and Shallice (1986), which is posteriorly integrated with the working memory model (Baddeley, 2003). In addition, the integration of semantic working memory with posterior and anterior areas of the cortex occurs with the activation of 10–14 Hz (Vernon et al., 2003), a frequency range close to the SMR.

The results from individuals in the SNF group, who underwent a sham training of neurofeedback, also presented with increases in their performances on the post-training DMTS, suggesting that the process of training is capable of inducing an effect on attentional processes, as reflected on their performance in the working memory task. Nonetheless, the comparison between subjects DMTS in the SNF group was not as significantly different as it was in the NF group.

Therefore, according to the principles of operant conditioning, the reinforcement must come in a contingent manner in order to provide conditioning. Even when the reinforcement is non-contingent, it is enough to influence the results because there is some feedback acting on individual actions (Strehl, 2014). Moreover, none of the participants considered themselves as part of a sham group, which meant the training was a repetition of their first training and the feedback was not provided according to what they actually did. Thus, the contingent expectation of the results of their actions was enough to interfere in the self-regulation ability of the individual (Witte et al., 2013). If the participant identifies the training as real and received feedback, even if non-contingent on their actions, they could make modifications for their own benefit and achieve improvements in their performance.

A study conducted by Reis et al. (2016) tested the effects of neurofeedback training on pre-frontal, central and parietal areas compared to working memory cognitive training. They demonstrated changes in cortical activity in the neurofeedback group as well as in the sham neurofeedback group, and alpha rhythm was enhanced in both groups. The neurofeedback group had a higher change in their alpha rhythm, but the sham group also changed, mainly through the attentional processes involved during the activity of neurofeedback. Their study suggested that neurofeedback was able to enhance the alpha rhythm at the frontal area of the cortex yet enhance an individual’s performance of working memory tasks when compared to cognitive training.

Working memory is a multicomponent model that involves more than one cortical area, mainly regions of medial temporal lobe and dorsolateral prefrontal cortex (D’Esposito and Postle, 2015). The EEG captures only activation at the superficial cortical layers III and V (Teplan, 2003), not providing information about deepest areas of the brain such as temporal medial lobe and inferior parietal cortex. Nonetheless, in this study it was possible to observe that the areas with greater activation during DMTS at the pre-training condition are those recruited during working memory tasks, that is frontal and central mostly in theta and alpha band (Klimgberg, 2010). It was observed a higher activation of alpha band at right hemisphere on interaction between experimental and placebo group, which is in accordance to Baddeley (2003) that higher activation at the right frontoparietal areas are linked to visual working memory content.

At the pre-training conditioning, it was observed a higher intensity activity and statistically differences in practically all measured regions between groups. However, in the post-training it can be observed changes in activity of those cortical areas, with less placement activation. That is, theta band activity was observed in all cortex during the pre-training condition, except at T3 and F3, nonetheless at the post-training less regions were active, with difference at frontal, temporal, central and occipital area. This pattern also happened to alpha band, in which all cortex was active during the task at the pre-training condition, except T3, and at the post training all the regions: frontal, temporal, central, occipital and parietal were active within less placement.

At the interaction between SNF and NNF, it was possible to observe a statically difference for gamma activation, at the condition pre and post-training. This activation in both condition for all subjects, reinforce that gamma band has an important role in the integration of connectivity during the working memory task (Constantinidis and Klingberg, 2016).

Regarding to the activation between pre and post training, the results demonstrated that NF group presented a lower activation in all frequency band between condition comparing to SNF. Whereas, the behavior result of NF was statistically significant in relation to SNF. Hence, it can be deduced that at NF group a less generalized activation was needed to ensure the working memory performance.

On the grounds that, modification in cognitive aging are evident by neurobiological changes, caused by volumetric differences in cortical structures, providing a less efficiency in the information processing including a diminishing in speed, working memory, inhibition and long-term memory (Park and Reuter-Lorenz, 2009), therefore the resources used by this population to counteract these challenges are the enhancement of activation during complexes tasks is to compensate, as observed by the SNF.

Reuter-Lorenz and Cappell (2008) named this compensation of *Compensation-Related Utilization of Neural Circuits Hypothesis—*CRUNCH, in which elderly will over activate the expected cortical area in order to keep the performance in a highly demand task, a strategy to overcome natural cortical changes. Therefore, the CRUNCH compensation might explain the generalized cortical activation observed in the SNF.

Belham et al. (2013) compared young and aging population in a working memory task observing a higher activation of theta at the central area for both, young and older, regarding to attentional and cortical integration of cognitive process, however the older participants presented greater activation at the beginning and middle of the task, correlating to CRUNCH in order to keep the performance.

On the other hand, for the NF group, the activation during task performance was less statistically significant as observed in the post-training spectrum, but the performance at DMTS was higher. Therefore, as the training was conducted in the central area and feedback was regard to increment of SMR, that favor attention and cortical integration (Kober et al., 2015) as a thalami-cortical inhibitory process, it can be inferred that the training induced neuronal plasticity by less activation during the task performance (Vermeij et al., 2017).

The neuromodulation of neurofeedback training relies on persistent human functional brain reorganization by neuroplasticity that is evidenced in cortical changes observed in post-training neuro-images (Chein and Schneider, 2005; Scholz et al., 2012; Ghaziri et al., 2013). These changes are based on a combination of Hebbian and homeostatic plasticity. This means that although the cellular activation by the stimulus condition leads to a long-term potentiation (LTP) of the firing neurons that enhances the postsynaptic and synaptic association, there is also a rebound to balance the likelihood of extremes such as excitation or inhibition (Sitaram et al., 2017).

The neuronal plasticity observed by NF training is in accordance to the INTERACTIVE model, that suggests activation is modified according to the characteristic of the training demand. That is, when a training involves task repetition, generally is associate with the diminish of activation, while a training involving metacognitive strategies and new learning might induce a higher activation at the expected area or activation of new areas (Belleville et al., 2014). The NF training in this study involved a reinforcement of the same region at the same frequency band during 10 sessions, characterizing as a repeated practice, in which there is a reduction of activation needed by the enhancement of efficiency at the region.

The study conducted by Naito and Satoshi (2014) evaluated the efficiency of recruited motor regions by FMRI in professional soccer players and other athletes. It has shown similar results according to the Interactive Model for repeated practice. Most of the athletes in the study have demonstrated lower activation of motor area when imaging their movements, with greater featured for the elite Brazilian soccer player Neymar that presented an even more reduced activation in relation to the others participants.

Moreover, the occipital region presented higher activation at post-training condition, which is in accordance with Sitaram et al. (2017) an area related to cognitive process of control during the NF training.

The working memory task were adapted using landscape images in order to have neutral content, however landscapes is associate to linguistic and emotional content that were not controlled during the study, therefore, some of the activation in temporal regions might be inferred as a codification of these content. Although, activation of theta-gamma coupling in frontal temporal regions are associate to visual working memory processing (Daume et al., 2017).

The SMR standard protocol applied at our study have not contemplate the motor artifact of facial muscle during NF training. That is, not controlled the alpha power that could interfere coupling SMR frequency during NF training. Therefore, those measures could be a confounder. Klimesch et al. (1994) proposed that the individualized alpha power is between the peak of alpha frequency and high alpha band presented by each individual and that alpha band may change with age. In our study the SMR frequency band was established between 12 Hz and 15 Hz and the alpha band 8–11 Hz, nonetheless as alpha band changes in aging brain waves it raises the question if there were alpha coupling during NFT that might interfere at the results (Bazanova and Aftanas, 2010). More research is needed to clarify this and any other possibilities.

In the present study, the increase in working memory occurred through effective neurofeedback training, although the sham group also showed an improvement. This demonstrates that exposure to the technique is sufficient to provide positive changes in the cognitive abilities of a healthy, aging population. The group that did not have any training had no difference in their performance, which reinforces the importance of the neurofeedback technique as a tool favoring the preservation of cognitive reserve (Brehmer et al., 2012).

## Conclusion

During the aging process, individuals experience changes in their neuropsychological abilities caused by neurobiological changes occurring over time. Consequently, techniques and tools that benefit the cognitive reserve become fundamental, as life expectancies have increased and individuals experience this stage of life for longer periods.

The present study has shown that neurofeedback training is a technique beneficial to preserve cognitive reserve in elderly populations. This comes as a result of an increase in the visual working memory capacity after neurofeedback training. Moreover, the behavioral results also demonstrate that the improvement of working memory occurred not only by the effective neurofeedback training but also in the sham group; therefore, exposure to the technique is enough to favor positive changes in the cognitive abilities of the individual.

The EEG spectrum power presented in the study reinforced activity expected in working memory tasks at frontal medial areas, likewise in frequency band, theta and alpha. It was also observed significant activation in gamma, reinforcing its role in integration of cognitive processing. Besides that, the observed differences in pre and post training for each group demonstrated a significant difference in the SNF, while in NF group has not change activation significantly, which can be explained by the interactive model-repeated training producing more efficiency in the recruited areas.

The study has some limitations. The sample size was small, especially the NNF group, which can bias the results, diminishing the reliability of the study. An increment of the sample size could favor an enhancement of the power in statistical analysis. In order to control the linguistic content of the landscape images of DMTS, the use of non-meaningful pictures or images from similar category could provide different cortical activities and mean of correct responses. As well as the use of data-based images with emotional content could favor the observation of this results.

Moreover, the neurofeedback protocol presented in the study have not controlled possible brain waves artifact provided by movement and imagination, as subject could use any strategy in order to achieve the training goal. Therefore, for future studies, the inclusion of the inhibition threshold to these frequency band (EMG and theta) in the protocol can control this interference in training.

Additionally, the EEG spectrum during the working memory task could have been associate with the event-related potential (ERP). Therefore, for future studies, this type of analysis could contribute in an experimental design to evaluate correct responses by task occurrence, image position and evoked potential.

Even though, with in limitation, the results pointed out that Neurofeedback is an accessible technique of neuromodulation using EEG with operant conditioning and cognitive self-perception that can favor the preservation of cognitive reserves in the elderly.

## Author Contributions

VCP, AG, ACPN and CT designed the study, analyzed the data and interpreted the data. VCP and AG collected the data. VCP, AG and CT wrote the article.

## Conflict of Interest Statement

The authors declare that the research was conducted in the absence of any commercial or financial relationships that could be construed as a potential conflict of interest.

## Footnotes

^1^http://sccn.ucsd.edu/eeglab
